# Within-Host Recombination in the Foot-and-Mouth Disease Virus Genome

**DOI:** 10.3390/v10050221

**Published:** 2018-04-25

**Authors:** Luca Ferretti, Antonello Di Nardo, Benjamin Singer, Lidia Lasecka-Dykes, Grace Logan, Caroline F. Wright, Eva Pérez-Martín, Donald P. King, Tobias J. Tuthill, Paolo Ribeca

**Affiliations:** 1 The Pirbright Institute, Ash Road, Woking, Surrey GU24 0NF, UK; antonello.di-nardo@pirbright.ac.uk (A.D.N.); lidia.lasecka-dykes@pirbright.ac.uk (L.L.-D.); gracie.logan@gmail.com (G.L.); caroline.wright@pirbright.ac.uk (C.F.W.); eva.perez@pirbright.ac.uk (E.P.-M.); donald.king@pirbright.ac.uk (D.P.K.); toby.tuthill@pirbright.ac.uk (T.J.T.); paolo.ribeca@pirbright.ac.uk (P.R.); 2 Department of Statistics, University of Oxford, Oxford OX1 3LB, UK; benjamin.singer@bnc.ox.ac.uk

**Keywords:** recombination, quasi-species, intra-host diversity, linkage disequilibrium

## Abstract

Recombination is one of the determinants of genetic diversity in the foot-and-mouth disease virus (FMDV). FMDV sequences have a mosaic structure caused by extensive intra- and inter-serotype recombination, with the exception of the capsid-encoding region. While these genome-wide patterns of broad-scale recombination are well studied, not much is known about the patterns of recombination that may exist within infected hosts. In addition, detection of recombination among viruses evolving at the within-host level is challenging due to the similarity of the sequences and the limitations in differentiating recombination from point mutations. Here, we present the first analysis of recombination events between closely related FMDV sequences occurring within buffalo hosts. The detection of these events was made possible by the occurrence of co-infection of two viral swarms with about 1% nucleotide divergence. We found more than 15 recombination events, unequally distributed across eight samples from different animals. The distribution of these events along the FMDV genome was neither uniform nor related to the phylogenetic distribution of recombination breakpoints, suggesting a mismatch between within-host evolutionary pressures and long-term selection for infectivity and transmissibility.

## 1. Introduction

The foot-and-mouth disease virus (FMDV) is a prototypical member of the genus *Aphthovirus*, family *Picornaviridae*, together with bovine rhinitis A virus, bovine rhinitis B virus, and equine rhinitis A virus [1]. It causes foot-and-mouth disease, an acute and highly contagious vesicular disease of domestic and wild artiodactyls, with economically devastating consequences [2]. Animals exposed to FMDV usually develop viraemia within a few days of exposure, while clinical signs usually last for 1 to 2 weeks. In some cases, the virus can persist for years in carrier animals. These persistent infections occur often in buffaloes and other species where the infection progresses in a subclinical form.

Encapsulated in a non-enveloped virion, the FMDV RNA genome (of ∼8.4 kb in size) is surrounded by an icosahedral capsid formed by four structural proteins (VP4 to VP1), with 10 further non-structural proteins (Lpro, 2A, 2B, 2C, 3A, 3B1, 3B2, 3B3, 3Cpro, and 3Dpol) encoded by ten non-capsid coding regions [3]. Structural proteins possess determinants for infections and immunity, whilst the non-structural proteins are responsible for genome processing (i.e., structural protein folding and assembly) and replication [3]. The polyprotein is encoded by a single open reading frame which is flanked by 5’ and 3’ untranslated region (UTR) regions [4]. The capsid is preceded by a leader (Lpro) polypeptide which cleaves itself from the polyprotein.

The high genetic variability of FMDV is a common feature of RNA viruses. As a result of the high substitution rates in FMDV genomes, seven immunologically distinct serotypes—O, A, C, Southern African Territories (SAT) 1, SAT 2, SAT 3, and Asia 1—are currently circulating worldwide [5]. FMDV lineages tend to diverge rapidly and spread into new areas, and hence the geographic structure of FMDV lineages includes three continental epidemiological clusters in Africa, Asia, and South America, which can be further classified into seven distinct virus pools. Multiple serotypes circulate in each pool and some countries share lineages originating from different pools [6].

The high mutation rate and genetic variability of the FMDV genome also generate rich intra-host dynamics [7]. Within a given host, FMDV sequences usually appear as a single quasi-species or viral swarm, i.e., a cloud of similar genotypes differing only by a handful of mutations. This is a typical pattern of genetic variability in organisms with high mutation rates, such as RNA viruses [8,9].

Recombination is one of the determinants of FMDV genetic diversity [10,11,12], with new recombinants appearing regularly in the literature (see for example [13,14,15,16]). Systematic studies have confirmed that recombination in FMDV is a relatively common event [11,17,18], with further evidence provided for intra-typic capsid recombination [19,20]. However, exhaustive studies have focused on phylogenetic recombination signals [18,21], while there has been little evidence of recombination at finer evolutionary scales. Yet, for recombination to occur at all, the only possibility is that it occurs within some hosts during co-infections by different FMDV strains. Almost no direct evidence of within-host recombination has been discussed in the past.

A previous analysis of an inoculation experiment in African buffaloes by some of the authors [22] revealed a large amount of within-host recombination in structural proteins. This was possible due to the inoculation and co-infection of two divergent but closely related viral swarms or quasi-species. Recombination rates were found to be higher in the early acute phase of the infection, while they were reduced by an order of magnitude in the carrier phase. Although the recombination rates in the capsid were lower than estimates derived for neighbouring regions of Lpro and 2A, the overall amount of recombination in the capsid was still very high (and comparable with the FMDV mutation rates), contrasting with the scarce evidence of recombination among structural proteins of FMDV strains.

This evidence prompted us to examine eight samples from FMDV-infected African buffaloes and cattle where the presence of multiple strains or quasi-species structure was suspected [23] as well as public sequences from Asian buffaloes [24]. Deep sequencing of the full FMDV genome in these samples allowed us to build the first genome-wide picture of the within-host recombination patterns of FMDV.

## 2. Materials and Methods

### 2.1. Deep Sequencing

FMDV field isolates (mostly derived from African buffalo *Syncerus caffer*) were provided by the Food and Agriculture Organization of the United Nations (FAO) World Reference Laboratory for Foot-and-Mouth Disease (WRLFMD), Pirbright. These samples represent a subsample of those analysed in a separate paper (Lasecka et al., submitted to this Special Issue) [25]. As a part of standard virus isolation protocol each strain was subjected to virus isolation in tissue culture (3–5 passages in primary bovine thyroid (BTY) cells or in baby hamster kidney (BHK) cells). Isolates were sequenced on an Illumina MiSeq™ machine using a modified version of a previously described PCR-free protocol [26]. Briefly, total RNA was extracted from clarified infected cell lysates using TRIzol Reagent (Thermo Fisher Scientific, Waltham, MA, USA) as per the manufacturer’s instructions. Any residual genomic DNA was removed using DNA-free DNA Removal Kit (Thermo Fisher Scientific) following the manufacturer’s protocol. After precipitation with 3 M sodium acetate and ethanol, 10 µL (containing from 1 pg to 5 µg) of RNA was used in a reverse transcription (RT) reaction as previously described [27] with the exception that, in addition to Random Hexamers (Bioline Reagents Ltd., London, UK), two primers (Rev6 and NK72, previously described [26]) were included in the first incubation step, and the final incubation step at 42 °C was carried out for 40 min. Second-strand synthesis was carried out using the NEBNext mRNA Second Strand Synthesis Module (New England Biolabs, Ipswich, MA, USA) following the manufacturer’s protocol and subsequent cDNA extracted by the addition of equal volumes of phenol:chloroform:isoamyl alcohol (Thermo Fisher Scientific) followed by 3 M sodium acetate/ethanol precipitation, as described in [27]. cDNA was quantified using the Qubit dsDNA HS Assay Kit (Thermo Fisher Scientific) as per the manufacturer’s instructions, and a cDNA library was prepared using the Nextera XT DNA Sample Preparation Kit (Illumina, San Diego, CA, USA) following the manufacturer’s recommendations. Sequencing was carried out on the MiSeq platform using Miseq Reagent Kit v2 (300 cycle) chemistry (Illumina).

A reference sequence for each sample was assembled using an in-house pipeline (Ribeca et al., in preparation) based on SPAdes [28] and custom software. Reads were mapped to the assembled FMDV sequence using GEM [29] version 3 with global alignment parameters. Single Nucleotide Polymorphisms (SNPs) were called using an approximation of the Bayesian calling approach described for SNAPE-pooled [30], which is suitable for higher read depths. Clustal Omega 1.2.4 [31] was used to align the assembled consensus sequences to a multiple nucleotide/codon alignment of seven prototype sequences, one for each serotype. Positions in the text are always relative to sequence O/UKG/1/24 (GenBank accession number: AY593829).

All other samples were from Asian water buffaloes (*Bubalus bubalis*) and were retrieved from the Short Read Archive accession SRP07971. Their sampling and sequencing protocol has been recently published in [24]. They were analysed with the same in-house pipeline used for the other FMDV field isolates.

### 2.2. Multi-Swarm/Quasi-Species Structure

We selected eight WRLFMD samples that showed a clear population structure in viral sequences, with two sufficiently divergent haplotypes present at intermediate frequency, similar to that found in [22]. Details about the frequency of this multi-swarm structure among the samples and their differentiation and genetic variability can be found in [23]. To select only quasi-species at intermediate frequencies, after inspection of the SNP frequency distribution we considered SNPs with frequency >0.1. We also required SNPs to have high read coverage (at least 1000 reads containing the two main alleles for the African samples, since the typical Poisson noise on counts is then less than 10% of the count). To confirm that there was a strong haplotype structure, we required that either the SNP frequencies be concentrated around a given value (with a ratio of standard deviation/mean for the sample not above 1/4, in order to reject fluctuations much larger than Poisson noise), or that a locally well-defined quasi-species structure across consecutive SNPs be present (i.e., the correlation in frequencies of SNPs at a distance of <70 bp be above 0.6). Finally, samples satisfying the above criteria were selected only if the nucleotide diversity was high enough (at least 30 such SNPs, corresponding to a nucleotide divergence of about 0.4% between haplotypes, which is both unlikely to be generated by intra-swarm variability or sequencing errors, and is necessary to get multiple SNPs in the same read). Statistics for the selected samples are shown in Table 1.

None of the Asian buffalo samples from [24] satisfied these criteria. The causes are the different (primer-based) protocol used to generate sequences, the lower coverage, and the fact that minor quasi-species in these samples are at low frequency (i.e., SNPs in these samples do not exceed a frequency of 0.2). Therefore, we analysed these samples separately and applied more relaxed criteria, considering all SNPs with frequency >0.1 and coverage >500 in all Asian samples as pertaining to putative intermediate-frequency quasi-species.

### 2.3. Inference of Recombination

**Figure 1:**
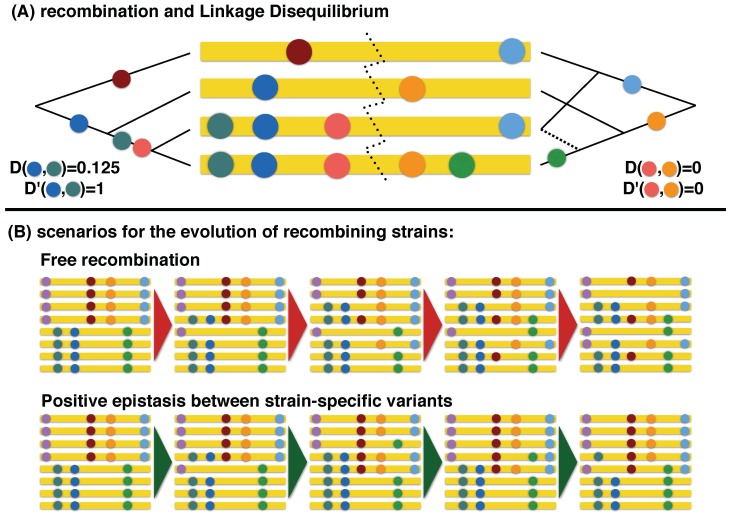
(**A**) Schematic illustration of the effect of a recombination event on sequences and SNPs. Each mutation occurs at most once along the tree. The left part of the sequence comes from a single genealogical tree and all variants are either strongly associated to each other or completely disjoint, hence there is complete linkage disequilibrium ($|D^{\prime }|=1$). The SNPs in the example have frequencies 0.5 and 0.75 and overlap in 50% of the sequences, hence *D* = 0.5 − 0.5 × 0.75 = 0.125. Since the overlap is also the largest possible one for these frequencies, max(D) = 0.125 and $D^{\prime }=1$. The recombination event in the middle of the sequence changes the genealogy (as can be be seen in the genealogical tree on the right, the event prunes the dashed branch and regrafts it elsewhere). Variants in the right part of the sequence are unrelated to the ones across the recombination event, i.e., the presence of one variant in a sequence is not informative about the presence of the other, and linkage disequilibrium is much reduced ($|D^{\prime }|<1$). In the example, the two variants both have frequency 0.5 but their frequency of overlapping is 0.25, hence *D* = 0.25 − 0.5 × 0.5 = 0. The maximum overlapping frequency would be 0.5, hence max(D) = 0.5 − 0.5 × 0.5 = 0.25 and $D^{\prime }=0$. (**B**) Schematic illustration of the two extreme scenarios for recombination, starting with a mixture of two strains. In the case of freely recombining sequences, the number of recombinants increases steadily with time. The inferred recombination rate corresponds to R=r·t where *r* is the recombination rates and *t* is the sampling time elapsed after the strains were mixed. In the epistatic case, if the fitness of the recombinants is 1−s times the fitness of the original strains, their number increases slightly until it reaches a stationary value determined by the recombination-selection balance and proportional to the ratio r/s. The inferred recombination rate at equilibrium corresponds to R≈r/s.

The classical population genetics statistics related to recombination is the linkage disequilibrium (LD), which is a measure of the association between alleles at two different SNPs [17]. LD is high when two variants are strongly associated, i.e., when the presence of a variant at a site in a sequence implies the presence or absence of the other variant at the other site, while it is close to 0 if the variants are randomly distributed across the sequences [32]. We estimated the linkage disequilibrium among pairs of SNPs in a sample using the normalised measure $D^{\prime }$. This measure is defined as $D^{\prime }=D/D_{max}$ if D>0 and $D^{\prime }=D/\left|D_{min}\right|$ if D<0, where $D=f\left(A_{1}A_{2}\right)-f\left(A_{1}\right)f\left(A_{2}\right)$ for two SNPs with ancestral alleles $A_{1}$ and $A_{2}$, while $D_{max}$ and $D_{min}$ are its maximum and minimum possible value given the frequencies of the variants [32]. These statistics are illustrated in Figure 1A.

For each pair of SNPs related to the quasi-species structure in a given sample, we computed $D^{\prime }$ from all the reads covering both sites. For this computation we considered only the two major alleles at each site. Without recombination, the value of $D^{\prime }$ is always ±1. Hence, $|D^{\prime }|<1$ is evidence of recombination, being equivalent to the so-called four-gametes rule [33], i.e., to the presence of all four major haplotypes (ancestral/ancestral, ancestral/derived, derived/ancestral, derived/derived). To estimate recombination rates, we selected only pairs of SNPs with $|D^{\prime }|<1$ and with each of the four major haplotypes appearing in >2 reads and in >0.2% of the reads. Inference of recombination rates was computed as $\hat{R}=-\log(|D^{\prime }|)$, following [22]. To find a minimum set of reliable recombination events, we selected SNP pairs satisfying the more conservative requirement of each major haplotype appearing in >5 reads and in >0.5% of the reads. The reason for such thresholds was to exclude as many artefacts as possible.

Several effects—sequencing errors, amplification errors, multiple mutations/backmutations in the same site—can result in possible artefacts, i.e., in values of $|D^{\prime }|<1$. To account for these effects, we compared our estimates with a conservative estimate of the background resulting from the presence of these effects. We take advantage of the fact that these effects do not only generate spurious recombinant haplotypes, but also other minor haplotypes that do not correspond to pairs of major alleles at the two sites. For example, if the haplotypes across the two sites were TC and CT, sequencing errors or multiple mutations would not only generate artifactual recombinants TT and CC, but also other minor haplotypes like CA, CG, and AT. In terms of abundance, CC and TT could still be favoured over the others, because of heterogeneities in mutation/error rates. To account for such differences in rates between different pairs of nucleotides, an excess factor could be estimated from ratios of counts such as #CC over (#CA + #CG). In turn, we can estimate these ratios by counting which mutations appear as low frequency variants (f<0.01) in the samples. The results suggested that a conservative estimate of this factor would be at most around 1.9.

To obtain a “background” estimate, for each pair of SNPs we replaced the count of one of the four major haplotypes with the count of all minor haplotypes differing from the major haplotype by one nucleotide at a given SNP. The idea is that if the major haplotype were a result of multiple mutations/sequencing errors at that SNP position, these minor haplotypes would have been generated by the same process. The choice of the haplotypes involved in this replacement is driven by maximisation of the difference in counts, and consequently of the “background” recombination rates. We also multiplied the count of minor haplotypes by a factor of 2 to account for possible heterogeneities in mutation/error rates discussed above. Then, we repeated the analysis on all pairs of SNPs. As an example, if a pair of SNPs were to have haplotype counts #TC = 10, #CT = 10, #CC = 2, #TT = 3, #CA = 1, #CG = 2, #AT = 1, we would replace #CC by the count of compatible minor haplotypes 2 × (#CA + #CG) = 6 that could be generated by mutations/errors in the second site. This would decrease the original value of D=f(TC)−f(T·)f(·C) from D≈0.15 to a “background” value of about 0.13 and would increase the corresponding estimate of recombination rate. Instead, if the haplotype counts were #TC = 10, #CT = 10, #CC = 2, #TT = 3, without minor haplotypes, we would replace #CC by 2 × 0, hence increasing the value of *D* to 0.16.

As a null comparison for statistical tests and normalisation, we consider a set of SNP pairs chosen with a distribution of read depth as similar as possible to the set of pairs where recombination was detected, containing 10 times the number of pairs of the latter.

### 2.4. Phylogenetic Inference of Recombination Rates

Recombination breakpoints were inferred from a collection of 376 full-genome FMDV sequences retrieved from GenBank (Supplementary Table S1) constituting a representative collection of the full-genome sequence variability from the National Center for Biotechnology Information (NCBI). The nucleotide alignment was inferred from an amino acid alignment of the polyprotein sequence obtained by Clustal Omega 1.2.4 [31]. Inference of recombination events was performed using RDP4 Beta 4.95 [34] by 100 bootstrap replicates and setting a window size of 200 bp and step size of 20 bp, with the highest acceptable probability of sequences sharing a recombinant region set at 0.05. The distribution of recombination breakpoints was generated by 1000 permutations setting a window size of 200 bp. We thus estimated local rates of recombination from the density of breakpoints in windows of 200 bp. To account for local biases in the inference of recombination breakpoints due to sequence similarity and other features, we simulated 50 recombination breakpoints every 200 bp among random pairs of sequences in the same alignment and counted the number of breakpoints that are actually detected by RDP4. Recombination events are proportionally more difficult to detect in regions that contain low numbers of breakpoints in the simulated analysis.

We then tested if the distribution of the pairs of recombinant SNPs was uniform or if it was distributed with the same pattern as the phylogenetic breakpoints. For this, we performed a multinomial test conditioned on the number of SNP pairs where recombination was detected, comparing these SNP pairs to the null sets described above. Since it is challenging to estimate how many recombination events occurred between each SNP pair in a robust way, but recombination appears to be low in all samples, we approximated the computation of the probabilities by assuming no more than a single recombination event between each pair. Probabilities for the multinomial test were assigned by summing the local rates of recombination (uniform or phylogenetic) over all bases between the SNP pairs.

## 3. Results

### 3.1. Recombination Events and Rates

**Figure 2:**
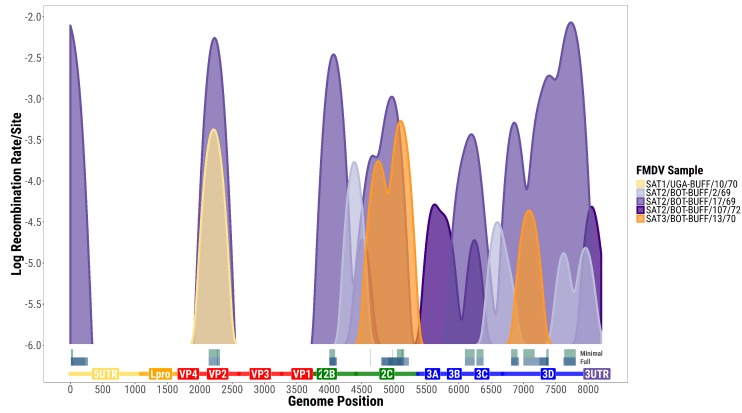
Foot-and-mouth disease virus (FMDV) recombination rates inferred from each sample. Samples are coloured according to their serotype and the corresponding rates are smoothed via a Gaussian kernel with a width of 200 bases. The grey intervals at the bottom illustrate the minimal recombination intervals (**top**) and the full set of recombining pairs of Single Nucleotide Polymorphisms (SNPs) (**bottom**).

After filtering, multiple samples (all collected from African buffaloes) revealed pairs of SNPs that show clear signals of recombination. We detected the presence of 27 such pairs in SAT2/BOT-BUFF/17/69, 3 pairs in SAT3/BOT-BUFF/13/70, and 1 each in SAT1/UGA-BUFF/10/70 and SAT2/BOT-BUFF/107/72. Note that these samples cover all the three SAT serotypes, although most of the recombining pairs are found in a single SAT2 sample. All these 32 recombining pairs could be explained by recombination inside a minimal set of 15 intervals. These intervals are illustrated in Figure 2.

The reliability of these results can be assessed by comparing the number of recombining SNP pairs detected in our data with the ones generated by the “background” procedure. We estimated that after filtering we would expect to observe a background of only one pair of artefactually recombining SNPs, versus the 32 pairs found in our sequences. Further proof that the signal was due to recombination events was given by the length of the intervals between recombining SNP pairs, which was larger than the one between non-recombining SNP pairs with similar coverage (mean: 114 bp versus 100 bp, median: 121 bp versus 93 bp). This behaviour is expected under recombination due to the higher chance of recombination in larger intervals; however it would not occur if the signal could be explained by multiple mutations or sequencing errors only, since they do not depend on the distance between SNPs.

The distribution of recombining SNP pairs was apparently inhomogeneous along the genome. It was significantly not uniform along the open reading frame (ORF) (multinomial test: p<0.01 for all pairs and <0.01 for minimal pairs). There is only one pair of recombining SNPs upstream of the ORF, and the low amount of recombination in structural genes can be clearly seen in Figure 2. The analysis in [22] suggested that recombination occurs in the capsid, but with rates reduced by a factor of 5–10 as compared to the neighbouring regions of Lpro and 2A. The results of the analysis presented here confirm that within-host recombination does occur in the capsid, but it is less abundant than in the flanking protein regions by about an order of magnitude. The “background” value is presented close to each estimate to show the expected extent of noise due to non-recombination factors.

An approximate estimate for the highest genome-wide recombination rate among the samples is about $10^{-4}$ recombination events per site per sequence since the quasi-species structure was formed, which is an unknown amount of time. Although we cannot easily infer the absolute recombination rates, the relative rates of recombination differed clearly between samples and can be more reliably estimated. The only samples that showed some significant genome-wide average rate of recombination per site (normalized by the highest value) are the ones in Table 2. Note that these estimates differed by two orders of magnitude between different samples. Local rates along the genome are shown in Figure 2, although the signal is quite noisy.

A few of the samples collected from Asian buffaloes [24] also reveal potential signals of recombination from different serotypes: a sample of serotype A contains 50 putative recombining SNP pairs (versus 19 for the “background” analysis) and other samples of serotype Asia1 contain 10, 7, and 6 putative recombining pairs (versus 3, 1 and 1 “background” pairs). This suggests that intra-host recombination is not uncommon and is not only a characteristic of SAT viruses, but can be found in other FMDV serotypes as well. It also suggests that African buffaloes are not the only species where intra-host recombination occurs, and that there is potential to observe these recombination events in cattle as well (even if both buffalo species are sub-clinical carriers, and the divergence between *Bubalus* and *Syncerus* is smaller than the divergence of cattle from any buffalo species).

### 3.2. Within-Host versus Phylogenetic Recombination

**Figure 3:**
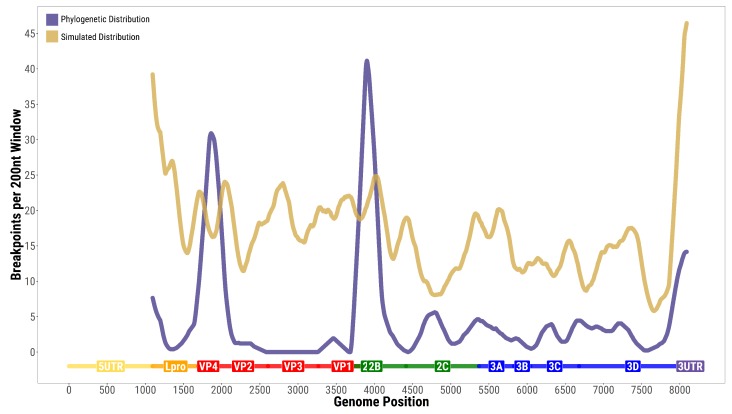
Phylogenetic signal of recombination along the nucleotide sequence of the polyprotein: Number of recombination breakpoints over a 200-bp window from RDP4 analysis of the real data (violet) and of simulated data (gold) obtained by adding 50 simulated recombination events per 200-bp window.

From the same samples, we also studied phylogenetic recombination (i.e., recombination occurring when one considers the evolution of transmitted strains circulating outside the host). The inferred phylogenetic pattern of genomic breakpoints is illustrated in Figure 3 and is similar to previous FMDV studies [18,21]. Two clear clusters of breakpoints are present on both sides of the capsid. The large number of recombination events in these regions suggests that capsid swapping is not uncommon in the evolution of FMDV genomes, similarly to other aphthoviruses [21]. The well-known cold spot corresponding to the capsid region [18,21] is also apparent in the analysis.

More interestingly, the distribution of within-host recombination events along the ORF sequence did not correspond to the distribution inferred at phylogenetic scales ($p<10^{-7}$ from the multinomial test, both for all pairs and for minimal pairs). The two distributions share some general features (e.g., low recombination in Lpro, hotspots at both sides of the capsid, coldspot inside the capsid, widespread recombination in non-structural proteins) but their fine-scale patterns and the relative weights of the hotspots are quite different.

**Figure 4:**
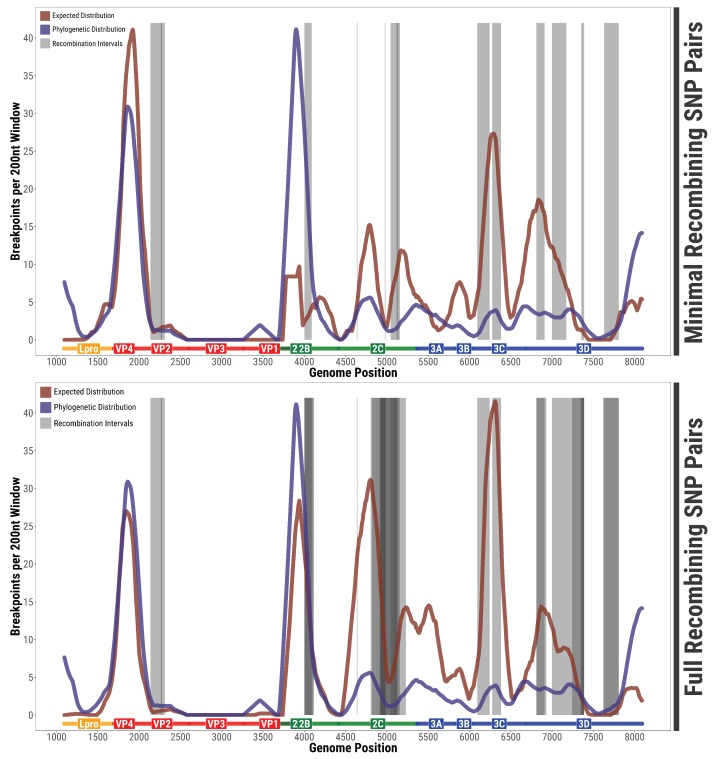
Comparison of within-host and phylogenetic recombination signal: (**Top**) Location of intervals between minimal recombining SNP pairs (grey) and expected distribution of recombination events corrected for the SNP distribution (red) along the FMDV genome. The expected distribution is based on the number of phylogenetic breakpoints inferred by RDP4 over a 200-bp window (blue) corrected for the probability of detecting a recombination events depending on the genomic location. (**Bottom**) Same plot for all recombining SNP pairs and the corresponding expected distribution.

An illustration of the difference between the actual position of recombining SNP pairs and the expected locations according to the phylogenetic density of breakpoints and the distribution of SNPs is shown in Figure 4. Among the most interesting differences, there was a surprisingly large number of within-host recombination events in some locations in non-structural genes, especially 2C and 3D. Even more interestingly, there seems to be a little unexpected hotspot of within-host recombination in the middle of the VP2 region, which does not correspond to the main phylogenetic hotspot of “capsid-swapping” recombination located around VP4.

This mismatch between recombination at different scales hints at the possibility that the evolutionary forces acting on recombinant sequences during intra-host evolution could be different from the ones involved in the long-term transmission and spread of the virus, as will be discussed in the next section.

## 4. Discussion

### 4.1. Impact of Selection against Recombinants

One of the assumptions of our estimates of recombination rates is that co-infection by multiple swarms occurs on a short timescale (i.e., the swarms either merged at some point in the chain of transmission leading to the infection, or they originated from two independent infections of the same animal) and that viral sequences are freely recombining within the hosts. In this case, the population evolves out of equilibrium and the number of recombinants increases in time (Figure 1B). The linkage disequilibrium between variants decreases accordingly: initially, the LD is at its highest possible value, but then it decreases in time as $D=D_{max}e^{-r\cdot t}$, where *t* is the time since the origin of the multi-swarm and *r* is the recombination rate for the interval between the SNPs.

However, these assumptions do not take into account selective pressures. In fact, linkage disequilibrium is also affected by epistasis, i.e., interactions between the fitness effect of different variants. Recombination breaks the association of variants present in the original swarms: if this association has a positive effect on fitness (i.e., if the original swarms have a higher fitness than their recombinants), the amount of recombinants tends to be reduced. Hence, epistatic interactions often act in opposition to recombination and cause an effective increase in LD [35,36].

In the most extreme case, the strength of selection against recombinants is strong enough to overcome recombination. In this case, the structure of the swarms reaches a stationary equilibrium with a constant fraction of recombinants (Figure 1B). For two equally abundant quasi-species with fitness 1 and assuming that single recombinants have fitness 1−s, the stationary fraction of recombinants is about 4r/s, provided that the fitness effect *s* is much larger than the recombination rate *r*. This can be derived as the selection-recombination equilibrium solution of the deterministic equations for selection and recombination between two loci [36]. If this were the actual scenario, then the quantity $\hat{R}$ that we measure would actually correspond to $\hat{R}=-\log(|D^{\prime }|)\approx r/s$ instead of the overall recombination rate r·t estimated for the case of free recombination.

The two scenarios are very different—the case of free recombination is of non-equilibrium dynamics, while the case of strong epistasis reaches a dynamic equilibrium—but, in both scenarios, the estimate based on LD is also an estimate of recombination (albeit with different coefficients). The difference between the two scenarios is that the selection coefficient *s*, i.e., the fitness cost of recombinants, could depend on the genomic region and therefore contribute to the observed patterns of genomic recombination.

In general, it is reasonable to assume that the actual intra-host dynamics lies between the two scenarios outlined above. In fact, at shown in the more detailed within-host study in [22], recombinants increase with time, suggesting an out-of-equilibrium scenario, but the observation of the effective long-distance increase in LD between the two mosaic blocks of VP1 hints at positive epistatic interactions between the quasi-species-specific variants. It is also reasonable to assume that the fitness of recombinants might depend on the genomic region where recombination occurred, and therefore contribute to the observed patterns of genomic recombination.

However, any such intermediate non-equilibrium scenario has important consequences for recombination at phylogenetic scales. In fact, even if recombination were to increase within each host, there must be a strong selection pressure against recombinants, since the final number of phylogenetic recombinants appears to be relatively small. This strong selection represents the actual bottleneck for the long-term viability of recombinants. By exclusion, such a selective pressure should act either during the infection process, or during the transmission process from one host to another. Hence, the long-term appearance of recombinants is more likely to depend on these selective pressures for infectivity and transmissibility than on the intra-host evolutionary dynamics.

Selection pressures for infectivity and transmissibility need not be related in any way to intra-host fitness, especially in the carrier state in buffaloes. A mismatch between these selective pressures and the genomic regions involved could be a plausible explanation for the mismatch we observed between within-host recombination and phylogenetic breakpoints.

### 4.2. Conclusions

In this paper, we presented the first genome-wide study of within-host recombination in FMDV. Our work does not attempt to make the case for the presence of extensive intra-host recombination for this virus, since overwhelming direct evidence for within-host recombination within the capsid region of SAT1 FMDV genomes was already presented recently in [22] from a complex dataset obtained from a long-term follow-up study conducted in artificially infected buffaloes. Here, we perform a genome-wide analysis of intra-host recombination rates obtained from a variety of samples across different virus types (SAT1, SAT2, SAT3, Asia1 and A) and hosts (African and Asian buffalo, while we were unable to find recombination in cattle).

The key finding of our study is that the recombination patterns we observed contrast with the distribution of breakpoints inferred by both previous phylogenetic analyses, and further phylogenetic analyses conducted in this paper on a larger set of FMDV genomic sequences. This result is consistent with the findings previously presented in [22], and suggests that the drivers at play during in-host evolution might be different from those shaping FMDV evolution at phylogenetic and population scales.

Recombination is a well-known evolutionary process in picornaviruses. Intra- and even inter-typic recombination plays an important role in the evolution and pathogenicity of enteroviruses, as exemplified by outbreaks of recombinants of vaccine-derived strains of polioviruses with coxsackieviruses [37]. Tissue culture experiments have shed light on the frequency and mechanisms of recombination in the poliovirus genome and in other enteroviruses [38,39,40,41]. However, we are not aware of any other study on the patterns of intra-host recombination in enteroviruses or other picornaviruses.

The role of within-host recombination is not yet fully understood. Intra-host recombination events could play a significant role in increasing genetic diversity in FMDV quasi-species [7,10]. In fact, while the low fidelity of the polymerase and the correspondingly high mutation rate are major drivers in generating new sequence variants, recombination could significantly increase the amount of different combinations of these variants. Note that both mutation and recombination are aspects of replication fidelity. The balance of mutation and recombination could also be relevant in alleviating the deleterious mutational load, as recently suggested in [42].

Recombination is not the only evolutionary force acting on FMDV swarms/quasi-species. Selection pressures act on genomic variants at different stages of the infection and transmission of the virus. However, not much is known about the extent of intra-host selection. Some evidence has been found [22] that epistasis and selection against recombinants play a role in the within-host evolution of VP1, and it has been suggested [18] that similar selective forces shape the mosaic structure of the FMDV genome at larger evolutionary scales. While we do not know the extent to which selection plays a role in modulating within-host recombination patters, our results, combined with those in [22], suggest that many recombinants could have a lower fitness during transmission and subsequent infections, while still surviving and replicating within hosts. In fact, we hypothesise that the fitness disadvantage due to reduced transmission and infection rates of recombinants is a plausible explanation for the mismatch between within-host and phylogenetic recombination patterns.

We do not know if recombination patterns could depend on the host species. A priori, we expect that these patterns would be mostly driven by features of the viral sequence and polymerase. However, it is worth noting that we were able to detect recombination in 5 out of 7 African buffalo samples, as well as in a handful of Asian swamp buffaloes, while no within-host recombination could be detected from the one cattle sample we have. This is hardly statistically significant; in addition, it could well be an artefact due to the lower divergence between co-infecting swarms in cattle [23] and the consequent challenges in detecting recombination. In fact, the lowest genetic divergence between swarms among our samples is found precisely in our only sample from cattle.

However, it should also be emphasised that recombination is closely related to the number of replications, since it requires co-infection of the same cell by multiple viruses and their replication. Increased replication rates are expected to result in increased recombination rates. Since buffaloes are subclinical carriers of FMDV and can harbour the virus for a longer time than cattle, it is also conceivable that the virus would replicate within this host for a longer time. As a result, the overall numbers of apparent recombination events would be much higher in carrier species than in other host species.

In addition to the host species, actual recombination rates per unit time might possibly depend on the viral serotype as well. Further studies will be required to understand if such differences exist. Unfortunately, under natural conditions such studies are possible only in the presence of a strong haplotype structure, which until now has been observed most often in SAT viruses circulating in buffaloes. Differences in recombination rates between serotypes (SAT1/2/3 versus Asia1, A, and O) could also be masked by the differences between buffalo hosts (African buffalo versus Asian swamp buffalo). A larger amount of samples from different serotypes and host species, including cattle, is needed to make further progress and identify the factors influencing within-host recombination rates.

The impact of the few passage of the isolates in tissue culture should also be clarified. Recombination occurs in FMDV cell culture [10] and could contribute to the rates measured here. Also, the selective pressure on the virus in tissue cultures is different from that within the host. This could affect the composition of the viral population and the fate of recombinants and other variants.

The samples presented in this study show relatively low amounts of recombination and variability. The combination of these features with the limitations due to the sequencing process and the short read lengths available caused some difficulties in detecting true recombination events above the noise due to sequencing errors and multiple mutations. In turn, these difficulties make a direct estimation of absolute recombination rates difficult. Although we were able to compare the patterns of genomic recombination, we cannot reliably estimate the relative strength of within-host and phylogenetic rates of recombination from the data presently available to us. Undoubtedly this will be addressed in future studies. Nevertheless, this paper suggests that better experimental protocols and deeper sequencing of a larger range of samples from infected animals should be able to shed a clear light on the genome-wide dynamics of recombination in FMDV, and on the intra- and inter-host evolutionary forces that shape the FMDV genome.

## Figures and Tables

**Table 1:** Average length for mapped reads (Avg Read Len), average coverage (Avg Cov), number of selected SNPs, and their average minor allele frequency (Avg Freq) for each sample. SAT: South African Territories.

Sample	Serotype	Host	Avg Read Len	Avg Cov	# SNPs	Avg Freq
SAT3/BOT-BUFF/13/70	SAT3	African buffalo	122	10,970	58	0.36
SAT1/UGA-BUFF/10/70	SAT1	African buffalo	120	7843	36	0.17
SAT2/BOT-BUFF/2/69	SAT2	African buffalo	114	9330	67	0.34
SAT2/BOT-BUFF/17/69	SAT2	African buffalo	119	9255	115	0.19
SAT2/BOT-BUFF/107/72	SAT2	African buffalo	128	4735	108	0.15
SAT1/TAN/22/2012	SAT1	cattle	160	11,639	31	0.17
SAT3/ZAM/P2/96 (NAN-11)	SAT3	African buffalo	165	3098	144	0.31
SAT3/ZIM/P6/83 BUFF-16	SAT3	African buffalo	100	1413	34	0.38
Samples from Ramirez et al. [24]	A, O, Asia1	Asian buffalo	see [24]	see [24]	see [24]	see [24]

**Table 2:** Average recombination rates per site in each sample, normalized with respect to the highest average recombination rate. Background values represent an estimate of the spurious signal due to sequencing errors or multiple mutations.

Sample	Recombination Rate	(“Background”)
SAT3/BOT-BUFF/13/70	0.1	(0.008)
SAT1/UGA-BUFF/10/70	0.06	(0)
SAT2/BOT-BUFF/2/69	0.011	(0.002)
SAT2/BOT-BUFF/17/69	1	(0.005)
SAT2/BOT-BUFF/107/72	0.018	(0.001)

## Supplementary Materials

The following are available online at http://www.mdpi.com/1999-4915/10/5/221/s1.
